# Validation of a Prediction Rule for the Diagnosis of Rheumatoid Arthritis in Patients with Recent Onset Undifferentiated Arthritis

**DOI:** 10.1155/2013/548502

**Published:** 2013-02-28

**Authors:** Zaida Bedran, Cristian Quiroz, Javier Rosa, Luis J. Catoggio, Enrique R. Soriano

**Affiliations:** Servicio de Clínica Médica, Hospital Italiano de Buenos Aires, Instituto Universitario Escuela de Medicina Hospital Italiano de Buenos Aires, and Fundacion Dr. Pedro M. Catoggio Para el Progreso de la Reumatología, Peron 4190, C1199ABB Buenos Aires, Argentina

## Abstract

*Objectives*. To validate van der Helm-van Mil score (vHvM) and new ACR/EULAR criteria for the diagnosis of rheumatoid arthritis (RA) in patients with undifferentiated arthritis (UA). *Patients and Methods*. Adult patients with UA (swelling ≥2 joints of less than 6 months duration, without diagnosis, and never treated with disease modifying drugs). *Results*. Ninety-one patients were included. Mean age: 55.6 years (SD: 17.4), 74% females. Median symptoms duration was 2 months (IR: 1–4 months). Mean van der Helm-van Mil score was 6.9 (SD: 2). After a mean followup of 6.2 months (SD: 6), 40.7% patients fulfilled ACR 1987 RA classification criteria, 28.6% fulfilled other diagnostic criteria, and 31% remained as UA. Receiver operator characteristic curve's (ROC's) area under the curve (AUC) for the vHvM score for diagnosis of RA was 0.83. A cutoff value of 6.94 showed sensitivity of 81% and 79.7% specificity. For the new ACR/EULAR criteria, the ROC AUC was 0.93, and a value equal to or greater than 6 showed 86.5% sensitivity and 87% specificity. *Conclusion*. van der Helm-van Mil prediction score and the new ACR/EULAR criteria proved to be valuable for the diagnosis of RA in patients with early UA.

## 1. Introduction

Rheumatoid arthritis (RA) is a systemic disease characterized by chronic inflammation that often leads to joint destruction. A greater awareness of RA [1, 2] has led to new efforts in order to establish a definitive diagnosis as early as possible after onset of symptoms [1–4]. Identifying patients with early arthritis at risk of developing persistent and/or erosive arthritis is mandatory for selecting a treatment strategy, according to the current early aggressive treatment approach [4]. The American College of Rheumatology (ACR) 1987 [5] classification criteria for RA were developed for clinical trials and research purposes, and it is not an appropriate tool for applying in the very early phase of the disease, mainly because of low sensitivity [6]. On the other hand, the majority of patients who present with recent onset arthritis have undifferentiated arthritis (UA) which is a form of arthritis that does not fulfill the classification criteria for a more definitive diagnosis. It is known that around 40%–50% of them may experience spontaneous remission, whereas RA develops in one-third of patients with UA [7, 8]. Therefore, finding predictors of the disease to take individualized decisions regarding treatment is one of the most important challenges in RA. According to the UA evolution mentioned previously, we believe that the consequences of over- or undertreatment of this disease are a keynote issue.

In the last decade, several developments have improved the capacity to recognize early RA. On the one hand, ultrasound (US) and magnetic resonance imaging (MRI) might improve our ability to detect inflammation at early stages [9, 10]. On the other, anticyclic citrullinated peptide (anti-CCP) antibodies identify patients with UA who have a significantly increased risk to meet ACR classification criteria for RA at a later evaluation [11–14]. At present, a precise model that predicts the disease course in patients with recent onset UA is lacking. Symmons et al. have stated that “no set of predictive criteria has been able to discriminate between individuals ultimately destined to developing RA and those not ” [15]. 

 In 2007, van der Helm-van Mil and collaborators developed a prediction model that predicts progression from UA to RA, using clinical variables that are easily assessed in clinical practice [16]. This score included simple items such as age, sex, distribution of inflamed joints, number of tender and swollen joints, morning stiffness, acute-phase reactants, and presence of rheumatoid factor and anti-CCP antibodies. This score works as an aid in addressing the problems of undertreatment (delayed treatment in patients with UA whose disease will progress to RA) and overtreatment (treatment with potentially toxic drugs in patients whose synovitis will remit spontaneously).

More recently, the American College of Rheumatology (ACR) and European League Against Rheumatism (EULAR) developed new criteria for the diagnosis of early RA [17, 18]. These new classification criteria have been described with the understanding that, at presentation, RA may be an evolving disease and that the final phenotype can be altered by therapeutic. It was designed to identify a subset of individuals who present with short duration of symptoms for whom the risk of symptom persistence or structural damage is sufficient to be considered for intervention with DMARDs.

These models have not been validated in our country, and the role of each one of their components in developing countries has not been established.

## 2. Objectives

The primary objectives of this study were to validate van der Helm-van Mil et al.'s prediction rule for the diagnosis of RA in a cohort of patients with early UA and to evaluate the contribution role and costs of each one of its components for the diagnosis of RA.

The secondary objective was to assess the usefulness of the new ACR/EULAR criteria for the diagnosis of RA in the same population.

## 3. Patients and Methods

### 3.1. Study Design

This was a cohort study of adult patients with early UA.

Consecutive adult (>18 years of age) patients attending the outpatient Rheumatology Unit at the Hospital Italiano de Buenos Aires, with early UA willing to participate (completing the informed consent), were included. Early UA was defined as swelling in 2 or more joints revealed on physical examination of less than 6 months of disease duration without a definite diagnosis and who had never been treated with any disease modifying antirheumatic drug (DMARD).

The following exclusion criteria were used:

- diagnosis of a definite rheumatic disease at first visit, according to the treating rheumatologist;
- fulfillment of 1987 ACR classification criteria of RA [5]; or fulfillment of criteria for another rheumatic disease;
- previous use of DMARDs;
- previous use (within onset of symptoms) or prolonged use of steroids (over 30 days).

### 3.2. Assessment

 At the first visit, the rheumatologist completed a questionnaire regarding the presenting symptoms as reported by the patient: type, localization, and distribution of initial joint symptoms, symptom duration, and course of the initial symptoms. The patient's smoking history and family history was also recorded.

Patients rated the severity of morning stiffness on a visual analog scale (VAS range 0–100 mm). Patients also completed the validated Health Assessment Questionnaire (HAQ) [19]. A 44-joint count for tender and swollen joints was performed. 

Baseline blood samples were obtained for determination of C-reactive protein (CRP), erythrosedimentation rate (ESR), the presence of IgM rheumatoid factor (RF), and the presence of anti-CCP, as determined by ELISA.

van der Helm-van Mil et al.'s score was calculated, once laboratory results were obtained, using clinical data at the first visit.

Patients were followed with a tight control approach for 6 months after inclusion into the study, or until a definitive diagnosis was established. At every followup visit, disease status was assessed to determine whether RA or another specific disease had developed, based on fulfillment of the 1987 ACR criteria.

van der Helm-van Mil et al.'s score and the ACR and the EULAR score were calculated for each patient. A receiver operating characteristic (ROC) curve was constructed to determine the best cutoff point for both scores and the area under the ROC curve calculated as a measure of the overall discriminative ability of both scores. Sensitivity, specificity, and positive and negative values and likelihood ratios (performance properties) with their 95% confidence intervals (CIs) were calculated for each score. For van der Helm-van Mil score, the best cutoff value showed by the ROC curve and a value of 8 points or greater were used for these calculations. For the ACR/EULAR score, a cutoff value of 6 was used. 

For cost calculation, charges to the patient's medical insurance were used without adjustment. Incremental cost effectiveness ratios for the complete van der Helm-van Mil prediction score and for the prediction score without including the anti-CCP antibodies and RF were calculated. Hospital prices for a clinical visit (accounting for the rheumatologist assessment of the score = 10 US dollars), CRP (1.8 US dollars), anti-CCP antibodies (62 US dollars), and RF (2 US dollars) were used as proxy of costs. The best cutoff value for the diagnosis of RA of each score was obtained from an ROC curve and used to calculate the percent of patients correctly classified, which was used as measure of effectiveness. 

Values were expressed as mean and standard deviation (SD) or median and interquartile range (IR). Categorical variables were expressed as proportions with their 95% confidence intervals (95% CIs). Continuous variables were compared with *t*-test or Mann-Whitney test, and categorical variables were compared with Chi-square test.

## 4. Results

One hundred patients were enrolled. Nine patients were excluded because of incomplete data (4 patients) or lost to followup (5 patients). Ninety-one patients were finally included until October 2010. Mean age was 55.6 years (SD: 17.4), and 67 (74%) were females.  Table 1 summarizes general characteristics of all patients included.

Median disease duration of symptoms at first visit was 2 months (IR: 1–4 months). Pattern of arthritis involvement at the beginning of the disease was polyarthritis in 44 (48%) and oligoarthritis in 47 (52%). Significantly more females never smoked, and more males smoked in the past (Table 1).

At first visit, patients were active with a mean number of tender and swollen joints of 6.8 (SD: 5.1) and 5.5 (SD: 4.2), respectively (Table 1), and acute-phase reactants were elevated in most patients. More patients were anti-CCP positive than rheumatoid factor (RF) positive (52% versus 28.6) (Table 1). In 23 of 47 patients (49%) with anti-CCP antibodies, RF was detected (*P* ≤ 0.0001).

Mean van der Helm-van Mil score was 6.9 (SD: 2). Thirty patients (33%) had a score equal to or greater than 8. Thirty-nine patients (43%) fulfilled at first visit the new ACR/EULAR criteria for RA using a cutoff value equal to or greater than 6.

Patients were followed until a definitive diagnosis was made, or they remained in followup as UA. Mean followup was 6.2 months (SD: 6) since first visit. Median followup was 4.2 months since first symptom (range 1 to 28 months). After a mean followup of 6.2 months (SD: 6), thirty-seven (40.7%) patients fulfilled ACR 1987 RA classification criteria. Another 26 patients (28.6%) fulfilled other diagnostic criteria (shown in Table 2), and 28 patients (31%) remained as UA (these patients were followed for a median of 8 (IR: 4–12) months). 

**Figure 1:**
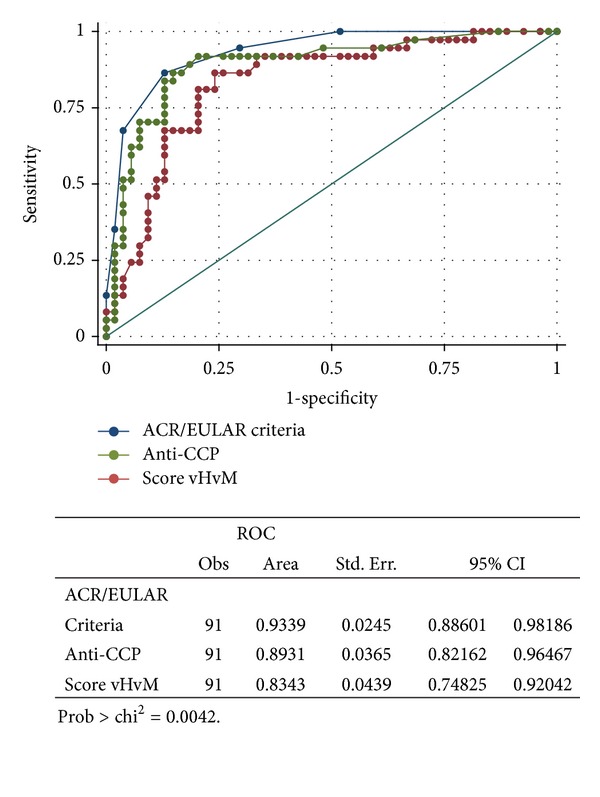
Comparison of receiver operator characteristic (ROC) curve for new ACR/EULAR criteria, van der Helm-van Mil prediction score, and anti-CCP antibodies for prediction of development of RA.

Receiver operator characteristic (ROC) curves for the van der Helm-van Mil prediction score for the development of RA showed an area under the curve (AUC) of 0.83 (95% CI: 0.75–0.92) (Figure 1). A cutoff point of 6.94 showed sensitivity of 81% with specificity of 79.7%, with positive LR of 3.98 and negative LR of 0.24. A prediction score equal to or greater than 8 had a sensitivity of 62.2% (95% CI: 44.8%–77.5%) and a specificity of 87% (95% CI: 75.10%–94.63%) for the diagnosis of RA (according to 1987 ACR criteria), with a positive predictive value of 76.7% (i.e., 23 of 30 patients with a score >8 showed progression to RA).

Anti-CCP antibodies showed an area under the curve of 0.89 (95% CI: 0.82–0.96) for the diagnosis of RA (Figure 1). 

Figure 1 also shows the ROC curve for the new ACR/EULAR criteria for the diagnosis of RA. The area under the curve was 0.93 (95% CI: 0.88–0.98). A cutoff value equal to or greater than 6 showed 86.5% sensitivity and 87% specificity for the diagnosis of RA.

In Figure 1, comparisons of receiver operator characteristic curves for van der Helm-van Mil prediction score, anti-CCP antibodies, and the new ACR/EULAR criteria are shown. The area under the curve was significantly better for the new ACR/EULAR criteria (*P* = 0.0042).

**Figure 2:**
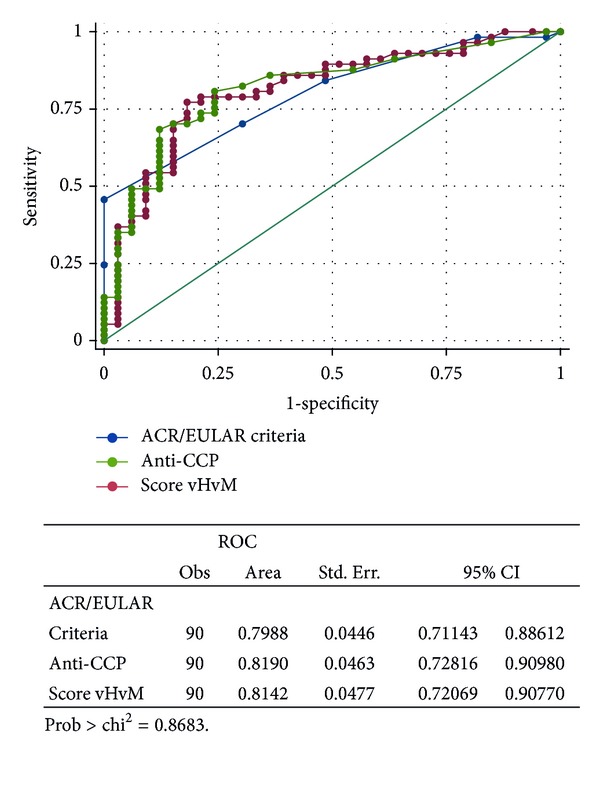
Comparison of receiver operator characteristic (ROC) curve of the new ACR/EULAR criteria, van der Helm-van Mil prediction score, and anti-CCP for prescription of DMARDs.

Figure 2 shows ROC curves for the comparison of the new ACR/EULAR criteria, van der Helm-van Mil prediction score, and anti-CCP antibodies for the prescription of DMARDs. The areas under the curve were lower than those for the prediction of development of RA. van der Helm-van Mil score seemed to work better for the prediction of prescription of DMARDs than the other scores, but the differences were not statistically significant (*P* = 0.8683).

Table 3 shows the relative risk of different variables associated with development of RA in univariate analysis. Rheumatoid factor, anti-CCP antibodies, and van der Helm-van Mil prediction score were significantly associated with development of RA, whereas elevated ESR, elevated DAS28 score, male sex, and symmetric involvement were not. 

Table 4 shows the logistic regression models for the diagnosis of RA including the prediction score (as a dichotomous variable with a cutoff value of 8), after adjusting for other variables not included in the score. A prediction score equal to or greater than 8 showed an OR of 18 (95% CI: 4.5–71.9; *P* = 0.000) and was the only variable significantly associated with development of RA. 

As shown in Table 5, adding anti-CCP antibodies to the clinical assessment and CRP portion of the prediction score produced an incremental cost of 344 US dollars for each additional patient correctly classified. Using the complete score produced an incremental cost of 337 US dollars over the score with only the clinical assessment and CRP, for each additional patient correctly diagnosed. 

## 5. Discussion

Ninety-one patients with undifferentiated early arthritis were included. The present study assessed the predictive accuracy of a prediction score that estimates the chance of progression to RA in an Argentine cohort of patients with early UA. We also evaluated the performance of the new ACR/EULAR criteria for the diagnosis of early RA. Disease duration was very short, reflecting that patients included represent real-life patients with very early UA. Surprisingly, half of patients were positive for anti-CCP antibodies. This might reflect that patients seen at the Rheumatology Unit represent a more selected population with severe disease. This is also shown by the elevated number of swollen joints, as well as high HAQ seen in these patients. 

Around 40% patients had an elevated van der Helm-van Mil score. Mean prediction score was higher than previously described in validation studies in Europe [20]. This might be explained by the high percentage of patients with anti-CCP antibodies included in our cohort and the fact that inclusion criteria required at least two swollen joints.

After a mean followup of nearly 6 months, around 40% patients fulfilled classic RA ACR criteria. This figure is similar to the one reported in other early UA cohorts and the one reported in the previous validation study [20]. 

The only individual variable significantly associated with development of RA was positive anti-CCP antibodies. Even after adjustment by other variables, the prediction score proved to be associated with development of RA in this cohort of UA. A cutoff value equal to or greater than 8 to predict development of RA showed a sensitivity of 62% and a specificity of 87%, similar to those reported in the validation study in Europe [20]. In our cohort, however, the best cutoff value was 6.94. 

The AUC for anti-CCP antibodies alone was even higher than the prediction score, suggesting a higher discriminative ability for the autoantibody alone than that provided by the complete score. This might be related to a low cutoff titer of anti-CCP used for the prediction score (20 units), thus increasing the number of positive samples. However, the best cutoff value for anti-CCP in the ROC curve was over 100 units. Anyway, the difference between both AUC was not statistically significant. The AUC for the prediction score for prescription of DMARDs was not very good, showing that it is not very useful to predict those patients that would receive therapy with disease modifying drugs. One explanation may be that antimalarials were considered as DMARDs, and almost all patients with the diagnosis of connective tissue diseases (Table 2) and many patients with UA received therapy with Hydroxicloroquine.

The new ACR/EULAR criteria performed even better than the van der Helm-van Mil prediction score and the anti-CCP antibodies. This information provides further support for using these new criteria for the diagnosis of early RA.

The simple incremental cost effectiveness analysis showed that the addition of anti-CCP antibodies and RF to the clinical assessment and routine laboratory test (PCR) was cost effective, with an ICER of only 337 US dollars for each additional patient correctly diagnosed.

In conclusion, van der Helm-van Mil prediction score [16, 20] and the new ACR/EULAR criteria proved to be valuable for the diagnosis of RA in patients with early UA in an Argentine cohort.

## Figures and Tables

**Table 1:** General characteristic of total population and by sex.

Characteristic	Total (*n* = 91)	Females (*n* = 67; 74 %)	Males (*n* = 24; 26 %)	*P* value (females versus males)
Age, mean (SD)	55.6 (17.4)	54.6 (18.1)	58.5 (15)	0.3459
Smoking status, *n* (%)				
Never smoked	61 (69.3)	49 (75.4)	(52.2)	
Smoked in the past	(21.6)	9 (13.8)	(43.4)	0.011
Still smoking	(9.1)	7 (10.8)	(4.4)	
HAQ, mean (SD)	1.1 (1.2)	1.2 (1.3)	0.9 (0.6)	0.3418
Number of tender joints, mean (SD)	6.8 (5)	6.6 (4.6)	7.4 (6.2)	0.4800
Number of swollen joints, mean (SD)	5.5 (4.2)	5.3 (3.8)	6.1 (5.1)	0.4089
Disease duration, (months) mean (SD)	3.6 (4.3)	3.9 (4.8)	2.9 (2.1)	0.3118
VAS pain, (mm) mean (SD)	59.9 (26.5)	58.9 (26.5)	62.7 (18.1)	0.5245
VAS patients global, (mm) mean (SD)	56.5 (25.6)	55.5 (26.6)	59.3 (23.1)	0.5427
VAS physician global, (mm) mean (SD)	41.6 (21.5)	41.3 (22.4)	42.4 (19.6)	0.8292
VAS morning stiffness, (mm) mean (SD)	49.8 (30.9)	49.5 (31.5)	50.5 (29.8)	0.8875
ESR, mean (SD)	36.9 (25)	35 (22.8)	42.3 (30.3)	0.2193
PCR, mean (SD)	15.7 (21.2)	13.1 (20.9)	21.8 (21)	0.1222
Positive RF: *n* (%)	27 (30)	21 (31)	6 (25)	0.559
Positive anti-CCP: *n* (%)	47 (51.6)	38 (57)	9 (37.5)	0.106
DAS28 (SD)	5.2 (1.2)	5.1 (1.2)	5.3 (1.2)	0.6708
van der Helm score, mean (SD)	6.9 (2)	7.1 (2.1)	6.1 (1.8)	0.0380
Mean ACR/EULAR criteria (SD)	5.1 (2.1)	5.1 (2.2)	5.1 (2)	0.9263

SD: standard deviation; HAQ: Health Assessment Questionnaire, VAS: visual analogue scale; ESR: eritrosedimentation rate; PCR: C-reactive protein; DAS28: disease activities score 28 joints,; RF: rheumatoid factor; CCP: cyclic citrullinated peptide.

**Table 2:** Diagnosis fulfilled by patients with early arthritis after followup.

Diagnosis	Number of patients	%
Rheumatoid arthritis	37	40.7
Undifferentiated arthritis	28	30.8
Polymyalgia rheumatica	4	4.40
Psoriatic arthritis	4	4.40
Osteoarthritis	3	5.26
Sjogren's Syndrome	2	2.20
Sarcoidosis	1	1.10
Other	12	13.18

Total	91	100

**Table 3:** Variables associated with devolvement of rheumatoid arthritis in univariable analysis.

Variable	Relative risk	95% CI	*P* value
Rheumatoid factor positive	3.96	1.85–8.46	0.0001
Anti-CCP antibodies positive	3.82	2.35–6.2	<0.0001
Score van der Helm-van Mil ≥8	4.8	2.3–10	<0.0001
DAS28 >5.1	1.01	0.65–1.6	0.9626
Symmetric involvement	0.99	0.67–1.45	0.9505
Male sex	0.73	0.35–1.52	0.3945
Older than 60 years	0.88	0.46–1.3	0.3739
Erythrosedimentation rate >40 mm	1.02	0.59–1.75	0.9382

**Table 4:** Logistic regression including van der Helm-van Mil score as a dichotomous variable.

Variable	Odds ratio	95% CI	*P* value
Age	0.95	0.93–0.99	0.049
Symmetric involvement	0.71	0.2–2.2	0.564
HAQ	0.85	0.4–2	0.710
ESR	0.99	0.96–1.02	0.545
DAS28	1.1	0.5–2.4	0.888
Disease duration (months)	0.99	0.9–1.1	0.934
vHvM score >8	17.99	4.5–71.9	<0.0001

ESR: erythrosedimentation rate; HAQ: Health Assessment Questionnaire; vHvM score >8: van der Helm-van Mil score equal or greater than 8; DAS28: disease activity score for 28 joints.

**Table 5:** Cost effectiveness ratio for complete van der Helm-van Mil prediction score, score without anti-CCP, score without RF, and score without anti-CCP and without RF.

Diagnostic score	Cost on 100 patients (US dollars)	Number of patients correctly diagnosed	Incremental cost	Incremental number of correctly diagnosed	ICER
vHvM without RF and without anti-CCP (cut off score >5.66)	1180	61	—	—	—
vHvM adding RF (without anti-CCP) (cut off score >6)	1380	64	200	4	50
vHvM adding anti-CCP (without RF) (cut off score >6.7)	7380	79	6200	18	344
complete vHvM (with anti-CCP and RF) (cut off score >6.94)	7580	80	6400	19	337

vHvM: van der Helm-van Mil prediction score; RF: rheumatoid factor; ICER: incremental cost effectiveness ratio.
